# Inhibitory Effects of *Parachlorella Beijerinckii* Extracts on the Formation of Advanced Glycation End Products and Glycative Stress-Induced Inflammation in an In Vitro Skin Dermis-Like Model

**DOI:** 10.1155/2022/8789903

**Published:** 2022-11-01

**Authors:** Yumi Imai, Yuya Nakashima, Toshihiro Kanno

**Affiliations:** Department of Research and Development, Chlorella Industry Co., Ltd., Hisatomi 1343, Chikugo, Fukuoka 833-0056, Japan

## Abstract

Advanced glycation end products (AGEs) are formed via a nonenzymatic glycosylation reaction called glycation. The formation and accumulation of AGEs increases in skin with age, contributing to the appearance of facial wrinkles and loss of skin elasticity. Therefore, inhibition of AGEs may delay skin aging. The microalgae *Parachlorella beijerinckii* has been used as a health food supplement for many years and contains carotenoids and vitamins that have antioxidant and anti-inflammatory effects. The aim of this study was to investigate whether *Chlorella* extract also has antiglycation activity. Antiglycation activity was measured using fluorescent AGEs, N*ε*-(carboxymethyl) lysine (CML), and N*ε*-(carboxymethyl) arginine (CMA) from glycated bovine serum albumin and type I collagen *in vitro*. A gel with a dermis-like structure consisting of collagen and a live fibroblast cell line was glycated with glyoxal. The content of fluorescent AGE, CML, and CMA, and the gel contraction activity were measured. In addition, to investigate the level of inflammation induced by the glycation of the collagen gel, the expression level of the receptor for AGEs and interleukin-8 were examined. Fat-soluble*Chlorella* extract suppressed the formation of fluorescent AGEs, CML, and CMA in both models. These results indicated that *Chlorella* extract directly inhibited AGE formation. The collagen gel contracted over time during culturing, whereas contraction was inhibited in the glyoxal-treated collagen gel. *Chlorella* extract remarkably attenuated the glyoxal-induced gel contraction. Moreover, *Chlorella* extract substantially decreased the fluorescent AGEs, CML, and CMA in the collagen gels with glyoxal. Glyoxal exposure increased the expression levels of interleukin-8 and receptor for AGE proteins in collagen gels, while *Chlorella* extract inhibited this increase. This study showed that fat-soluble*Chlorella* extract has a direct inhibitory effect on AGEs and decreases receptor expression for AGE-mediated inflammation by reducing AGEs. *Chlorella* may delay skin aging by inhibiting the formation and accumulation of AGEs.

## 1. Introduction

Protein glycation is a nonenzymatic reaction between reducing sugars and free amino groups in proteins that forms a reversible Schiff base. The Schiff base spontaneously rearranges into an Amadori product, which induces further oxidation, giving rise to advanced glycation end products (AGEs). In the skin, an increase in autofluorescence with age reflects the accumulation of fluorescent AGEs. N*ε*-(carboxymethyl) lysine (CML), a typical nonfluorescent AGE, as well as fluorescent AGEs, have been shown to be positively correlated with chronological age [1, 2]. Moreover, the accumulation of these AGEs is linked to an increase in facial wrinkles and loss of skin elasticity [3, 4].

Dermal extracellular proteins are considered major targets for glycation. Collagen is the main structural protein in the dermis, and its glycation induces the cross-linking of proteins. Accumulation of glycated collagen results in a long half-life and slow renewal of proteins *in vivo* [5]. Moreover, AGEs adversely affect dermal homeostasis [6]. For example, accumulation of AGEs into the dermis layer inhibits the proliferation [7], accelerates apoptosis and senescence of fibroblasts [7–9], and impairs extracellular matrix (ECM) synthesis [10]. These biological responses interfere with the normal maintenance of the dermal structure. Inhibition of the maintenance of skin homeostasis by AGEs is considered to be dependent on enhanced receptor for AGEs (RAGE) signaling, which is a proinflammatory process. Ligands binding to RAGE results in the upregulation of RAGE expression, thereby perpetuating cellular inflammatory responses [11]. Therefore, inhibiting the formation and accumulation of AGEs and attenuating RAGE expression may be effective for maintaining skin homeostasis and suppressing skin aging.

Synthetic inhibitors, such as aminoguanidine (AG), have been used to inhibit the formation of AGEs and to strongly inhibit glycation. However, prolonged use of AG has been reported to cause some side effects [12]. Glycation increases with age and glycation products are considered to accumulate over several years [1, 2]. Therefore, prolonged glycation inhibition is vital to suppress their accumulation. Natural plant-derived glycation inhibitors are considered to have fewer side effects and are safer than synthetic products, allowing their prolonged use. Therefore, many plant-derived components have been studied for their ability to inhibit AGE formation worldwide [13–18]. These components have antioxidant and anti-inflammatory effects, which also exhibit inhibitory activity on the formation and accumulation of AGEs. *Chlorella*, a unicellular green alga, has been used as a health food supplement for many years and has been considered to be safe. It contains a wide variety of vitamins and carotenoids, which have recently been reported to have antioxidant, anti-inflammatory, and antidiabetic effects [19–21]. We have already reported that *Chlorella*–a *Parachlorella beijerinckii* strain–has inhibitory effects against obesity, dementia, muscle atrophy, and inflammasome activation in rodent models [22–25]. These inhibitory effects have been shown to be the synergistic effects of multiple carotenoids contained in *P. beijerinckii*. These findings suggest that *P. beijerinckii* has the potential to suppress the formation and accumulation of AGEs and AGEs-induced inflammation. However, no studies have evaluated the antiglycation activity of *P. beijerinckii*.

The purpose of this study is to examine whether the components of *P. beijerinckii* exhibit direct antiglycation activity by inhibiting the glycation of bovine serum albumin (BSA) and collagen by glucose and glyoxal using an in vitro model. We also determined the inhibitory effects of glyoxal-induced glycative stress in a dermis-like model constructed from collagen gel and live fibroblasts and evaluated its potential contribution to the suppression of skin aging.

## 2. Materials and Methods

### 2.1. Chemicals and Reagents

D-glucose, aminoguanidine hydrochloride (AGH), 40% glyoxal solution, chloroform, 2-propanol, and 70% ethanol were purchased from Wako Pure Chemical Corporation (Osaka, Japan). BSA was purchased from Sigma-Aldrich (St. Louis, MO, USA). Acid-soluble bovine skin collagen type I was purchased from Nippi (Tokyo, Japan).

### 2.2. Preparation of *Chlorella* Extract (CE)

*Chlorella* extract was prepared according to a previous method [25]. Briefly, *Chlorella* (*Parachlorella beijerinckii*) was cultured, dried, and powdered by Chlorella Industry Co. Ltd. *Chlorella* powder was extracted with methanol and chloroform (1 : 2) at 25°C overnight in the dark. The extract was filtered through a filter paper, evaporated under reduced pressure, and dissolved in ethanol. For saponification, 50% KOH (w/v) was added to the extracted solution for 2 h. After saponification, 3% NaCl (w/v), distilled water, and diethyl ether were added. The residue was extracted with diethyl ether three times. The upper layers were evaporated and dissolved in tetrahydrofuran, dimethyl sulfoxide, and ethanol (1 : 1 : 2) to prepare a stock solution. The stock solution was stored in the dark at −80°C. Carotenoids and tocopherol in Chlorella extracts were measured using high performance liquid chromatography (HPLC) as described in a previous method [25]. The HPLC analysis was performed by the Japan Food Research Laboratories. The extracts had the highest concentration of lutein (429.3 mg/g extracts), followed by *β*-carotene (97.7 mg/g extracts), zeaxanthin (40.3 mg/g extracts), *α*-tocopherol (27.8 mg/g extracts), and *α*-carotene (12.5 mg/g extracts). However, approximately 40% of the extract weight was unknown.

### 2.3. *In Vitro* Glycation Assay

BSA was dissolved in phosphate buffer (0.1 M, pH 7.4) with glucose or glyoxal. Glyoxal is a physiological metabolite formed by lipid peroxidation, ascorbate autoxidation, oxidative degradation of glucose, and degradation of glycated proteins. It has also been identified as a reactive dicarbonyl formed during AGE generation and is far more reactive than D-glucose and most other reducing sugars [26]. The glycation of BSA and collagen was carried out in accordance with a literature method [27–32] with some modification. For the glucose glycation test, 10 mg/mL BSA was incubated with glucose (0.5 M) at 37°C for four weeks [27, 28]. For the glyoxal glycation test, 2 mg/mL BSA was incubated with 20 mM glyoxal at 37°C for one week [29, 30]. CE treatment concentrations in the all experiments were chosen based on lutein in CE, the most abundant carotenoid in the extract. Concentrations of 1.65 and 16.5 *μ*g/mL CE (each contained 1 and 10 *μ*M of Lutein) were used for experimentation. AGH solution (1 mM) was used as a positive control [28]. Collagen was dissolved in phosphate buffer (0.1 M, pH 7.4) with glucose or glyoxal. For the glucose glycation test [31], collagen (2 mg/mL) was incubated with 100 mM glucose in phosphate buffer with or without CE (1.65 and 16.5 *μ*g/mL) at 37°C for four weeks. For the glyoxal glycation test, collagen (1 mg/mL) was incubated with 1 mM glyoxal in phosphate buffer with or without CE (1.65 and 16.5 *μ*g/mL) at 37°C for one week [32]. Glucose-BSA and glucose-collagen samples were collected at zero, two, and four weeks. Glyoxal-BSA and glyoxal-collagen samples were collected at zero and one week. Samples were kept at −80°C until analysis.

### 2.4. Determination of Fluorescent AGE Formation

Fluorescent AGEs, the irreversible products at the end stage of nonenzymatic glycation, were determined using a spectrofluorometer (Infinite® 200 PRO, TECAN, Grödig, Austria) at excitation and emission wavelengths of 355 and 460 nm, respectively.

### 2.5. Determination of CML

The OxiSelect CML competitive ELISA kit was purchased from CELL BIOLABS Inc. (San Diego, CA, USA). The concentration of CML was determined using an enzyme-linked immunosorbent assay (ELISA) according to the manufacturer's instructions. The absorbance of the samples was compared with that of the CML-BSA standard provided in the assay kit.

### 2.6. Contraction Capacity Study and CE Treatment Using an In Vitro Dermis-Like Skin Model

Dermis-like structure collagen gels were prepared by modifying previously described methods [33–35] using skin normal diploid fibroblasts (TIG-118 (JCRB0535)) and collagen. In brief, the gelation collagen solution was prepared using 1080 *μ*L of type I collagen (3 mg/mL) dissolved in 5 mM acetic acid, 198 *μ*L of 10× Hanks' solution, and 522 *μ*L of 5 mM acetic acid. Then, NaOH was added dropwise to neutralize the solution. The gelation collagen solution (1800 *μ*L in total) was mixed with fibroblasts (3.0 × 10^5^), resuspended in 200 *μ*L of fetal bovine serum and seeded into a 6-well culture plate, and the gels were detached from the rim of the wells and incubated at 37°C and 5% CO_2_ further for three days. The gels were photographed using a SONY DSC-WX350 camera (Sony Corporation, Tokyo, Japan). Gel areas were calculated using ImageJ software (National Institutes of Health, MD, USA).

We previously reported that the ingestion of *Chlorella* increased plasma lutein concentration to about 1.2 *μ*M [36]. Therefore, in experiments using a collagen gel dermis-like model, we considered the maximum concentration of the extract to be applied to the culture medium as 1.65 *μ*g/mL CE (1 *μ*M lutein). To examine the effect of CE on contraction capacity, fibroblasts used for collagen gelation were pretreated with CE (0, 0.165, and 1.65 *μ*g/mL) or AGH (50, 2000 *μ*M) for three days. Following this, they were treated again with the same concentration of CE or AGH. Then, to induce glycation, glyoxal (final concentration 400 *μ*M) was added and the cells were cultured for three days. In this experiment, AGH was examined at two concentrations, with the lowest concentration (50 *μ*M) being the blood concentration when 0.3 g of AG was ingested [37]. Thus, we designed this study using both low AG and high CE concentrations based on their actual blood concentration levels after oral administration.

### 2.7. Western Blotting Assays

The sodium dodecyl sulfate–polyacrylamide gel electrophoresis (SDS-PAGE)-separated proteins were electrophoretically transferred to a polyvinylidene difluoride (PVDF) membrane (Immobilon, Merck, Darmstadt, Germany). The membranes were blocked (Blocking One, Nacalai Tesque, Kyoto, Japan) and then incubated with the following primary antibodies: anti-CMA (AGE-M04, Cosmo Bio, Tokyo, Japan), anti-RAGE (sc-365154, Santa Cruz Biotechnology, Dallas, TX, USA), and anti-*β*-actin (20536-1-AP, Proteintech, Rosemont, IL, USA). Horseradish peroxidase-conjugated secondary antibodies (SA00001-1 and SA00001-2, Proteintech) were used. Detection was performed by chemiluminescence using enhanced chemiluminescence (ECL) Western Blotting Detection Reagents (GE Healthcare, Little Chalfont, UK Life Science, Chicago, IL, USA) and Western Lightning Ultra (Perkin Elmer, Waltham, MA, USA). The detected CMA and RAGE band intensities were analyzed and quantified using the Image Studio™ analysis software (LI-COR Biosciences, Lincoln, NE, USA).

### 2.8. AGE Extraction from Glycated Collagen Gels

Collagen gels were cut into small pieces with scissors. HCl (0.01 M) was added to the fragmented collagen gel to make a 100 mg/mL collagen solution (w/v). The solution was incubated overnight at 37°C and centrifuged at 15000 rpm for 5 min. AGEs were recovered from the supernatant. CML, CMA, and RAGE in the supernatant samples were analyzed using CML ELISA and western blotting. Samples collected from incubation were loaded onto gels after heating with 4× SDS sample buffer at 96°C for 5 min. Electrophoresis was conducted using 10% gels.

### 2.9. RNA Extraction and Quantitative Reverse Transcription Polymerase Chain Reaction (qRT-PCR) of Glycated Collagen Gels

To examine cell-mediated contraction, the gels were detached from the rim of the wells and incubated for up to 24 h. The gels were recovered and 1 mL ISOGEN (Nippongene, Tokyo, Japan) was added to glycated collagen gels. Then, the gel was cut with scissors and homogenized using a BioMasherII (Nippi, Tokyo, Japan). Total RNA was extracted from cells in the glycated collagen gels using ISOGEN in accordance with the manufacturer's instructions. RNA samples were reverse transcribed using the PrimeScript^TM^ RT Reagent Kit (TAKARA, Shiga, Japan), in accordance with the manufacturer's instructions. Additionally, qRT-PCR analysis was performed using specific primers (Table S1) and TB Green Premix Ex Taq II (Tli RnaseH Plus) (TAKARA). *β*-Actin was used as the reference gene. Gene expression was determined by qPCR using a thermal cycler (StepOne Plus; Applied Biosystems, Waltham, MA, USA). Relative quantification was performed using the ^ΔΔ^C_t_ method in combination with reference genes for data normalization.

### 2.10. Statistical Analysis

All experiments were performed in triplicate or greater. All graphs display the mean ± standard deviation (SD). Statistical analysis was performed using the Bell Curve for Excel (Social Survey Research Information Co., Ltd. Tokyo, Japan). Values of *p* were calculated using unpaired two-tailed Student's *t* tests. Differences between the groups were considered statistically significant at *p* < 0.05.

## 3. Results

### 3.1. Effects of CE on Fluorescent AGE Formation from an *In Vitro* Glycation Assay

**Figure 1:**
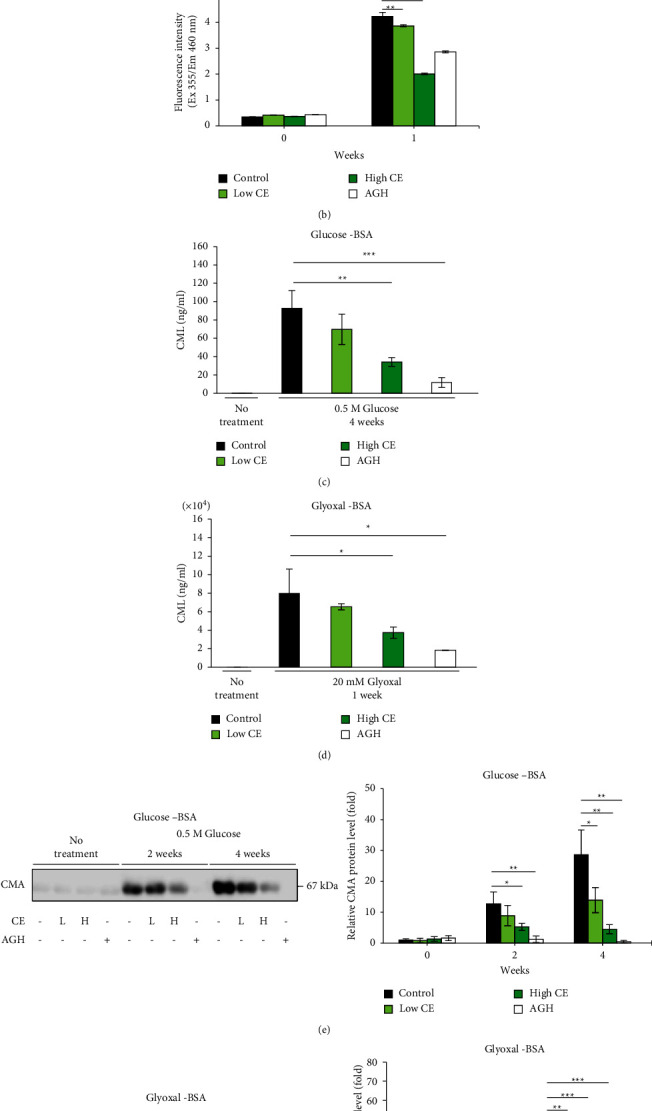
Effects of *Chlorella* extract on the formation of advanced glycation end products (AGEs) in a BSA glycation system. Bovine serum albumin (BSA) was incubated with glucose at 37°C for two or four weeks and with glyoxal for one week. Fluorescent AGEs (a, b; *n* = 4), N*ε*-(carboxymethyl) lysine (CML) (c, d; *n* = 4), and N*ω*-(carboxymethyl) arginine (CMA) (e, f; *n* = 3) were measured. *Chlorella* extract (CE) (1.65 *μ*g/mL) and high CE (16.5 *μ*g/mL) concentration were administered prior to incubation in each solution. An aminoguanidine hydrochloride (AGH) (1 mM) solution was used as the positive control. Each value represents the mean ± standard deviation (SD).  ^*∗*^*p* < 0.05,  ^*∗∗*^*p* < 0.01,  ^*∗∗∗*^*p* < 0.001 compared to control solution.

**Figure 2:**
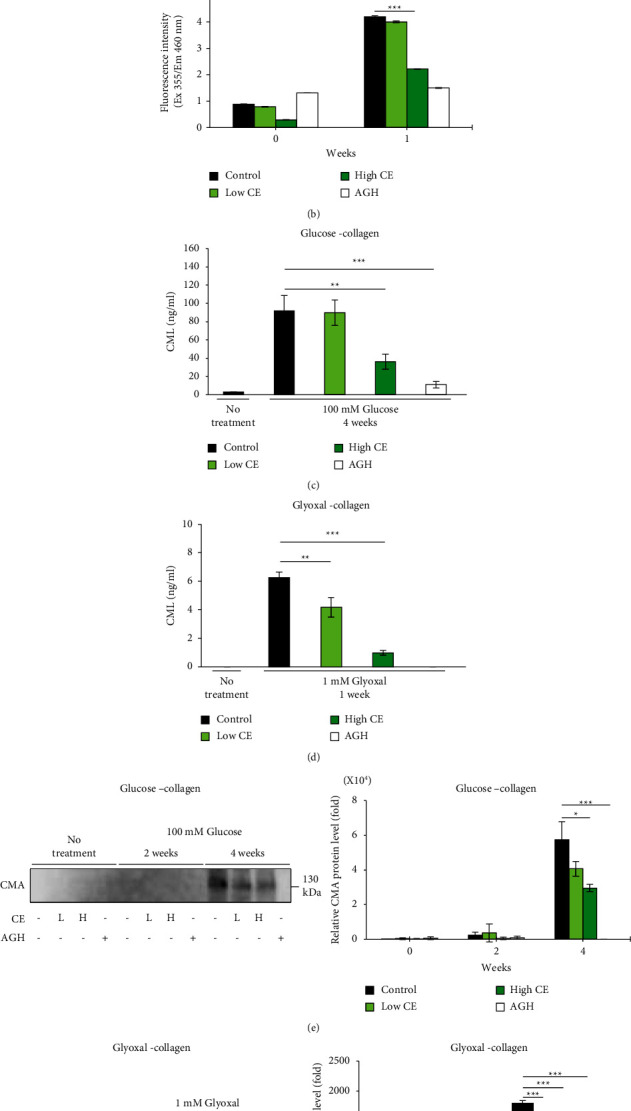
Effects of *Chlorella* extract on AGE formation in a collagen glycation system. Collagen was incubated with glucose at 37°C for two or four weeks and with glyoxal for one week. Fluorescent AGEs (a, b; *n* = 3), CML (c, d; *n* = 3), and CMA (e, f; *n* = 3) were measured. Low CE (1.65 *μ*g/mL) and high CE (16.5 *μ*g/mL) concentrations were administered prior to incubation in each solution. An AGH (1 mM) solution was used as the positive control. Each value represents the mean ± SD.  ^*∗*^*p* < 0.05,  ^*∗∗*^*p* < 0.01,  ^*∗∗∗*^*p* < 0.001 compared to control solution.

We prepared fat-soluble and water-soluble crude extracts of *Chlorella* and investigated their inhibitory effects on fluorescent AGE formation in an *in vitro* system. The fat-soluble extract greatly inhibited fluorescent AGE formation, whereas the water-soluble extract resulted in slight inhibition (Figures 1(a) and 1(b) and Figures S1(a)-S1(b)). The results showed that the antiglycation activity of *Chlorella* was contained in the fat-soluble component; therefore, the fat-soluble CE was used in all subsequent experiments. The formation of fluorescent AGEs was monitored every two weeks to determine the fluorescence intensity of the glucose-BSA and glucose-collagen solutions. The fluorescence intensity increased over time in both solutions. Solutions containing CE also showed an increase in fluorescence intensity over time. However, this was substantially suppressed when compared with that in the control solution (Figures 1(a) and 2(a)). Next, glucose was replaced with glyoxal and incubated with BSA and collagen solutions to measure the fluorescence intensity. As with the results for glucose, the fluorescence intensity of these solutions increased, and CE suppressed the increase in fluorescence of both solutions (Figures 1(b) and 2(b)).

### 3.2. Effects of CE on Nonfluorescent AGE Formation from an *In Vitro* Glycation Assay

CML and CMA are typically used as biomarkers for AGE formation. CML and CMA levels increased after glycation in the glucose-BSA and glucose-collagen solutions. The solutions containing CE also showed an increase in CML and CMA levels when compared with that in the control solutions. However, the increase was considerably suppressed when compared with that in the control solutions (Figures 1(c), 1(e), 2(c) and 2(e)). Glucose was replaced with glyoxal and incubated with BSA and collagen solutions to measure CML and CMA. As with the glucose results, the CML and CMA of these solutions increased after glycation; however, CE suppressed the increase in CML and CMA in both solutions (Figures 1(d), 1(f), 2(d), and 2(f)). These results indicated that CE directly inhibited both fluorescent and nonfluorescent AGEs in the *in vitro* glycation assay.

### 3.3. Attenuating Effect of CE on the Inhibition of Glyoxal-Induced Collagen Gel Contraction

**Figure 3:**
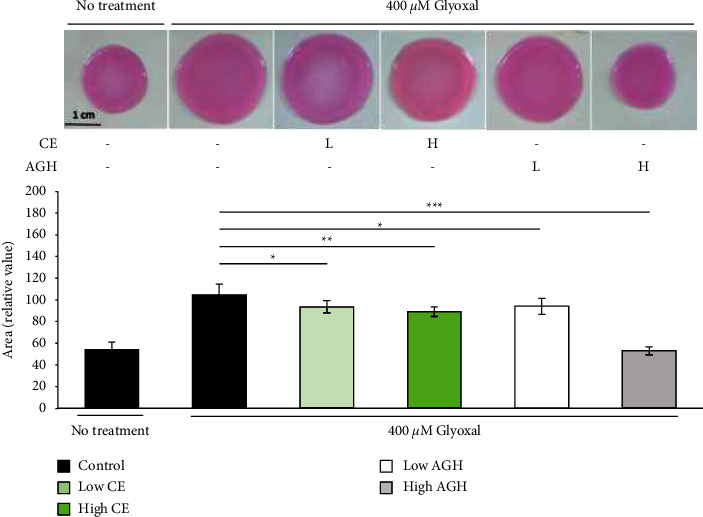
Effect of *Chlorella* extract on the contractile capacity of fibroblasts. Collagen gels were incubated for three days with 400 *μ*M glyoxal. Low CE (0.165 *μ*g/mL) and high CE (1.65 *μ*g/mL) concentrations were administered. AGH was used as a positive control at low (50 *μ*M) and high (2000 *μ*M) concentrations. Each value represents the mean ± SD (*n* = 6).  ^*∗*^*p* < 0.05,  ^*∗∗*^*p* < 0.01,  ^*∗∗∗*^*p* < 0.001 compared to glyoxal-treated control gel.

We examined cytotoxicity against fibroblasts used for *in vitro*dermis-like skin model. No cytotoxic effects were observed at the concentration of CE used in the experiment (Figure S2). We co-cultured fibroblasts and type I collagen to create a dermis-like collagen gel and investigated the effect of CE on gel contraction. The contraction of the collagen gel was induced by culturing for three days. When glyoxal-treated collagen gel was used, the contraction was inhibited, and the area expanded by about two times (Figure 3). However, when CE was added to the glyoxal-treated collagen gel, inhibition of contraction by glyoxal was remarkably attenuated in a dose-dependent manner when compared with that in the control (Figure 3).

### 3.4. Inhibitory Effect of CE on the Formation of AGEs in Glyoxal-Treated Collagen Gels

**Figure 4:**
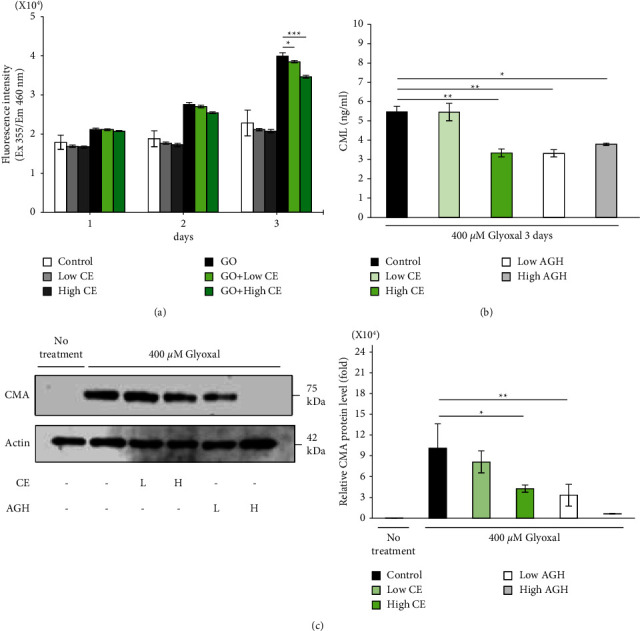
Inhibitory effects of *Chlorella* extract on the formation of AGEs in glyoxal-treated collagen gels. The collagen gel was incubated with glyoxal for three days. Fluorescent AGEs ((a) *n* = 3), CML ((b) *n* = 3), and CMA ((c) *n* = 3) were measured. Low CE (1.65 *μ*g/mL) and high CE (16.5 *μ*g/mL) concentrations were administered in fluorescent AGE studies. Low (0.165 *μ*g/mL) and high (1.65 *μ*g/mL) concentrations of CE were administered in CML and CMA studies. AGH was used as a positive control and administered at low (50 *μ*M) and high (2000 *μ*M) concentrations. Each value represents the mean ± SD.  ^*∗*^*p* < 0.05,  ^*∗∗*^*p* < 0.01,  ^*∗∗∗*^*p* < 0.001 compared to glyoxal-treated control gel.

We measured the fluorescence of the collagen gel as well as the levels of extracted CML and CMA. At the concentration of glyoxal that inhibited contraction activity (400 *μ*M) (Figure 3), the fluorescence of the gel itself increased only slightly (data not shown). Therefore, 10 mM glyoxal was used in the experiments on fluorescent AGEs in the collagen gel. The addition of CE to the collagen gel did not suppress the increased fluorescence on the first and second day after treatment with glyoxal, but considerably suppressed the increase in fluorescence on the third day (Figure 4(a)). In addition, formation and accumulation of CML and CMA in the gel were observed after treatment with 400 *μ*M glyoxal but were remarkably inhibited by treatment with CE (Figures 4(b) and 4(c)). These results indicated that CE inhibited both fluorescent and nonfluorescent AGE accumulation in collagen gels induced by glyoxal glycation.

### 3.5. CE Exerts an Anti-Inflammatory Effect by Suppressing RAGE Expression

**Figure 5:**
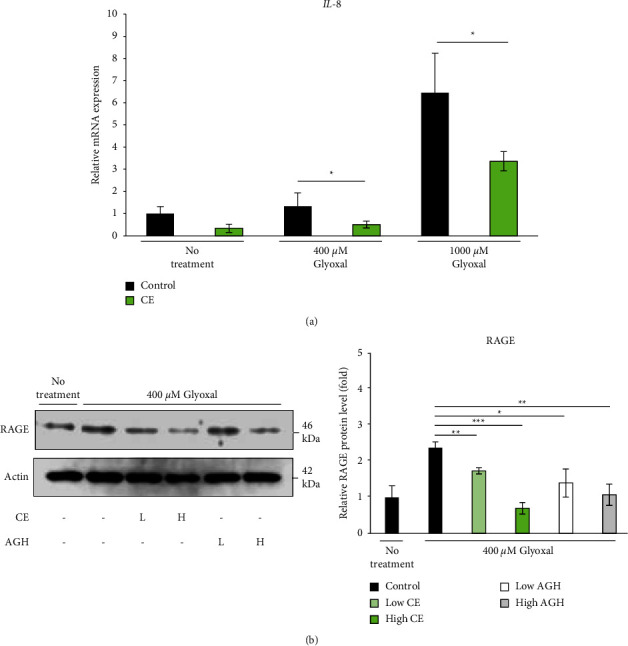
Effect of *Chlorella* extract on *IL-8* and RAGE expression in the glycated collagen gels. The collagen gel was incubated with 0, 400, or 1000 *μ*M glyoxal for 24 h and *IL-8* expression ((a) *n* = 3) was measured using real-time polymerase chain reaction (RT-PCR). CE was administered at 1.65 *μ*g/mL. The collagen gel was incubated with 400 *μ*M glyoxal for three days, and RAGE expression ((b) *n* = 3) was determined using western blotting. CE was administered at low (0.165 *μ*g/mL) and high (1.65 *μ*g/mL) concentrations. AGH was used as a positive control and administered at low (50 *μ*M) and high (2000 *μ*M) concentrations. Each value represents the mean ± SD.  ^*∗*^*p* < 0.05,  ^*∗∗*^*p* < 0.01,  ^*∗∗*^*p* < 0.001 compared to the glyoxal-treated control gel.

As shown in Figure 4, the accumulation of AGEs was confirmed after glyoxal treatment of the collagen gel. Therefore, to investigate the enhancement of inflammatory signals in collagen gels by AGEs, we measured the mRNA levels of proinflammatory chemokines and cytokines (interleukin-8 (*IL-8*), *IL-6*, and tumor necrosis factor alpha (*TNF-α*)). One day after glyoxal treatment of the collagen gel, the expression level of *IL-8* mRNA was substantially increased in a dose-dependent manner (Figure 5(a)). However, no increase in *IL-6* or *TNF-α* mRNA expression levels was observed (Figure S3). The increase in the *IL-8* mRNA expression level by glyoxal treatment was suppressed by the addition of CE (Figure 5(a)), which was consistent with the decrease in AGEs by CE (Figure 4).

We investigated the effect of CE on RAGE protein expression levels in collagen gels. RAGE expression increased by approximately 2.5-fold after treatment with glyoxal, but the addition of a high concentration of CE (16.5 *μ*g/mL) suppressed RAGE expression to the same level as that of the control group (Figure 5(b)).

## 4. Discussion

Our findings provide evidence that *Chlorella* is a new antiglycation agent that may be useful against skin aging. It acts by two independent mechanisms: direct inhibition of the formation of AGEs and suppression of RAGE-mediated inflammation.

In many studies, *in vitro*protein-glucose or protein-glyoxal antiglycation assay models have been used as the first step in testing the antiglycation activity of various ingredients [13–18]. Using the same model, we prepared water- and fat-soluble crude extracts of *Chlorella* powder and examined their antiglycation effects. Consequently, we found that the fat-soluble extracts had higher antiglycation activity than the water-soluble extracts (Figures 1 and S1). Zheng et al. compared the antiglycation activities of several *Chlorella* strains and found antiglycation activity in the fat-soluble portions [38], which is consistent with our results. In addition, astaxanthin, a carotenoid from *Chlorella*, has been found to strongly inhibit protein glycation [39]. Because we used the crude, fat-soluble extract, we could not identify the ingredients responsible for the antiglycation activity in this study. Moreover, the fat-soluble extract used in our experiments did not contain astaxanthin but contained several other carotenoids (lutein, zeaxanthin, *α*-carotene, *β*-carotene). These carotenoids may have synergistic effects that exhibited antiglycation activity in this study. However, given that approximately 40% of the components of fat-soluble*Chlorella* extracts are unknown, further research is required to identify the ingredients of *Chlorella* that exert antiglycation activity.

The formation of AGEs involves molecular processes based on simple or complex multistep reactions, including an oxidative step [40]. In this study, *Chlorella* suppressed the formation of CML and CMA, which passed the oxidation step during production. Furthermore, the *Parachlorella beijerinckii* strain that we used in this study has been shown to have antioxidant effects [23–25], suggesting that the antioxidant activity of this *Chlorella* extract might contribute to the inhibition of AGE formation. In addition, collagen type I is the major structural component of the dermal extracellular matrix and accounts for >70% of skin dry weight, providing the dermis with tensile strength and stability [41]. AGEs have been reported to accumulate in human and mouse skin tissues with age [1, 2, 42]. As CE also inhibited the formation and accumulation of collagen protein glycation (Figure 2), if fat-soluble components of *Chlorella* are able to reach the skin tissue, glycation and aging in the skin may be reduced.

In this study, we used aminoguanidine (AG) as a positive control for assessing antiglycation activity. AG is known to have strong antiglycation activity [43] and showed a higher inhibitory effect on glycation than the high concentration of CE in all experiments (Figures 1 and 2). Although AG has high antiglycation activity, it is not approved as a drug owing to its side effects [12]. *Chlorella*, more specifically *Parachlorella beijerinckii*, has been used as a dietary supplement in Japan for about fifty years and no side effects have been observed. Its continuous ingestion for 6−16 months in animal experiments or for 1−5 months in clinical trials was also not associated with adverse effects [23, 24, 36, 44–47]. Because the accumulation of AGEs in the skin occurs over many years, long-term ingestion is desirable for prevention. Thus, *Chlorella* may be used as a long-term ingestible antiglycation supplement as it has no reports about side effects. As a high CE concentration was shown to be as effective as or better than a low AG concentration in all experiments using collagen gel, it may show the same inhibitory effect on glycation as AG *in vivo*. However, because these experiments were conducted *in vitro*, further *in vivo* studies are warranted.

AGEs not only alter the physicochemical properties of biomolecules, such as proteins, lipids, and nucleic acids, but also bind to RAGE, initiating a cascade of signals that influence the cell cycle and proliferation, gene expression, inflammation, and ECM synthesis [11]. In this study, we found that *Chlorella* attenuated AGE-induced dysfunction in gels composed of skin fibroblasts and collagen (Figure 3). These results may be attributed to the direct inhibition of AGE formation and accumulation by *Chlorella*, as shown in Figures 1 and 2. Although the direct effect of *Chlorella* on glycation inhibition was not stronger than that of AG, *Chlorella* showed the same effect as AG in the experiments using collagen gel. This suggests that *Chlorella* has a mechanism to suppress the adverse effects induced by AGEs other than the direct inhibition of glycation. Owing to the fact that the lipophilic extract of *Chlorella* exhibits anti-inflammatory effects [25], *Chlorella* may suppress inflammatory signals in the fibroblasts contained in the collagen gels. Additionally, *IL-8* expression was reported to be induced via signaling pathways activated by RAGE ligand recognition in normal skin fibroblasts [48]. We found that *Chlorella* reduced the mRNA expression level of *IL-8*. These results suggest that *Chlorella* has at least two mechanisms for alleviating the adverse effects of AGEs. The first is direct AGE inhibition, and the second is suppression of the inflammatory pathway initiated by RAGE activation. RAGE gene and protein expression are upregulated by AGEs [48, 49], and their increased expression leads to chronic inflammation, which correlates with skin aging [50]. Therefore, regulation of RAGE expression is an important factor in delaying the process of skin aging. In this study, we found that *Chlorella* not only suppresses inflammation but also suppresses the expression of RAGE. These results indicate that *Chlorella* may delay skin aging by suppressing chronic inflammation. However, our study did not clearly indicate whether the antiglycation and anti-inflammatory effects of *Chlorella* inhibit skin wrinkles and loss of elasticity; therefore, further research is required regarding the inhibitory effects of *Chlorella* on these processes.

## 5. Conclusions

In this study, we demonstrated that carotenoids–the major fat-soluble extract of *Parachlorella beijerinckii*, which has been used as a safe supplement for many years–directly inhibit the glycation of BSA and collagen, as well as maintain the normal contraction of derma-like collagen gel by suppressing the accumulation of AGEs and RAGE-mediated inflammation. These results suggest that *Parachlorella beijerinckii* inhibits glycation stress and may slow age-related skin wrinkles and elasticity loss that occurs with an increase in age.

## Data Availability

All relevant data are available and can be provided upon request by the corresponding author.

## Supplementary Materials

Supplementary Materials Supplementary Table 1. List of primer sequences used for RT-PCR. Supplementary Figure S1. Effects of water-soluble Chlorella extract (WSE) on the formation of AGEs in BSA glycation. Supplementary Figure S2. Effects of Chlorella extracts on cell viability and cell death. Supplementary Figure S3. Effect of Chlorella extracts on interleukin 6 (IL-6) and tumor necrosis factor alpha (TNF-*α*) expression in the glycated collagen gels.
