# Supplementary material for: Inhibitory Effects of Parachlorella Beijerinckii Extracts on the Formation of Advanced Glycation End Products and Glycative Stress-Induced Inflammation in an In Vitro Skin Dermis-Like Model
Source: Evid Based Complement Alternat Med. 2022 Nov 1;2022:8789903. doi: 10.1155/2022/8789903 (PMC9643057; doi:10.1155/2022/8789903)
Supplement: Supplementary Materials — Supplementary Table 1. List of primer sequences used for RT-PCR. Supplementary Figure S1. Effects of water-soluble Chlorella extract (WSE) on the formation of AGEs in BSA glycation. Supplementary Figure S2. Effects of Chlorella extracts on cell viability and cell death. Supplementary Figure S3. Effect of Chlorella extracts on interleukin 6 (IL-6) and tumor necrosis factor alpha (TNF-α) expression in the glycated collagen gels. [file 8789903.f1.docx]

**Supplementary Materials**

**Supplementary Table 1.** List of primer sequences used for RT-PCR

| Primer name | Sequence 5’→3’ |
| --- | --- |
| β-actin Forward | CCACGAAACTACCTTCAAC |
| β-actin Reverse | GATCTTCATTGTGTGCTGGG |
| *IL-8* Forward | AGAGTGATTGAGAGTGGACC |
| *IL-8* reverse | ACTTCTCCACAACCCTCTG |
| *IL-6* Forward | TACCCCCAGGAGAAGATTCC |
| *IL-6* reverse | AGTGCCTCTTTGCTGCTTTC |
| *TNF-α* Forward | CGAGTGACAAGCCTGTAGCC |
| *TNF-α* reverse | TGAAGAGGACCTGGGAGTAGAT |


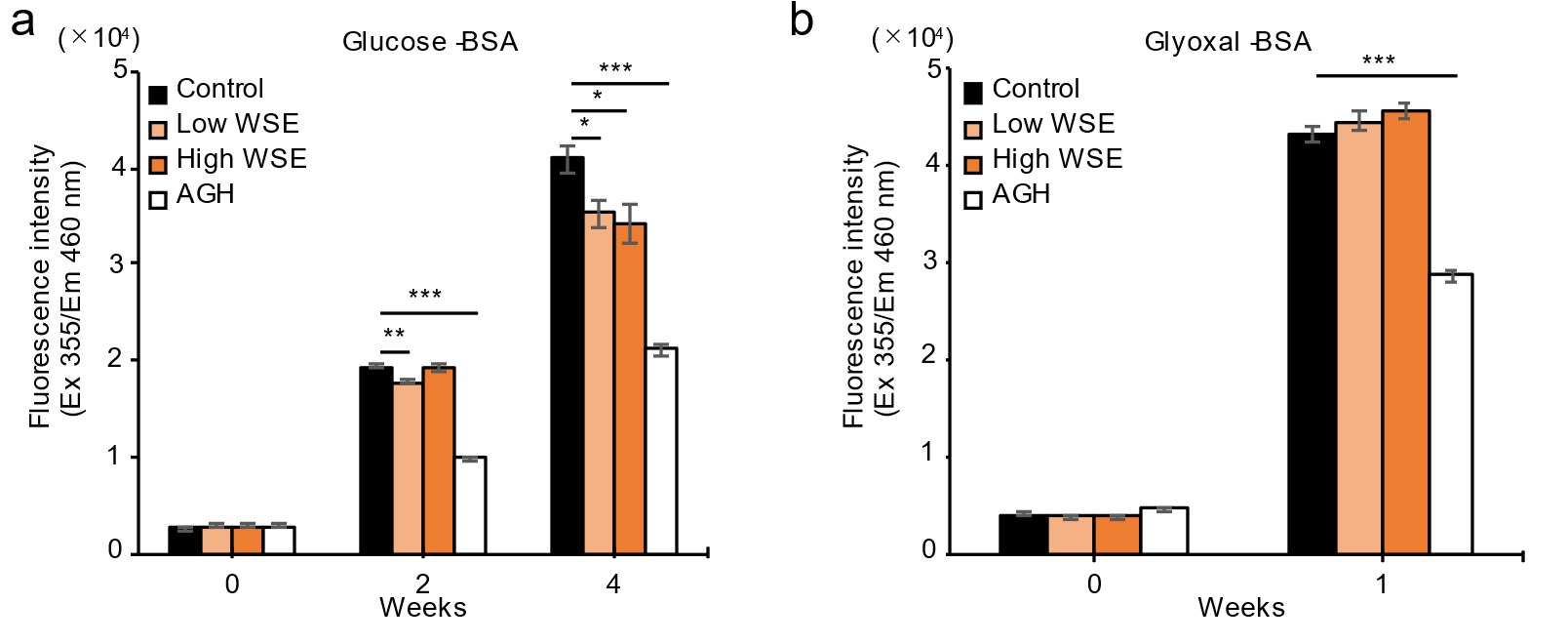


**Supplementary Figure S1.** Effects of water-soluble *Chlorella* extract (WSE) on the formation of AGEs in BSA glycation. WSE was prepared using *Chlorella* powder. *Chlorella* powder (0.5 g) was extracted using ultrapure water (50 mL) at 100°C for 25 min. Centrifugation was performed at 3000 rpm for 15 min, then the supernatant was filtered through a 0.2 μm membrane. BSA was incubated with glucose at 37° C for two or four weeks and with glyoxal for one week, and fluorescent AGEs (a, b; n = 3) were measured. WSE was administered at low (1.65 μg/mL) and high (16.5 μg/mL) concentrations prior to incubation in each solution. An AGH (1 mM) solution was used as the positive control. Each value represents the mean ± SD. **p* < 0.05, ***p* < 0.01, ****p* < 0.001 compared to control solution.


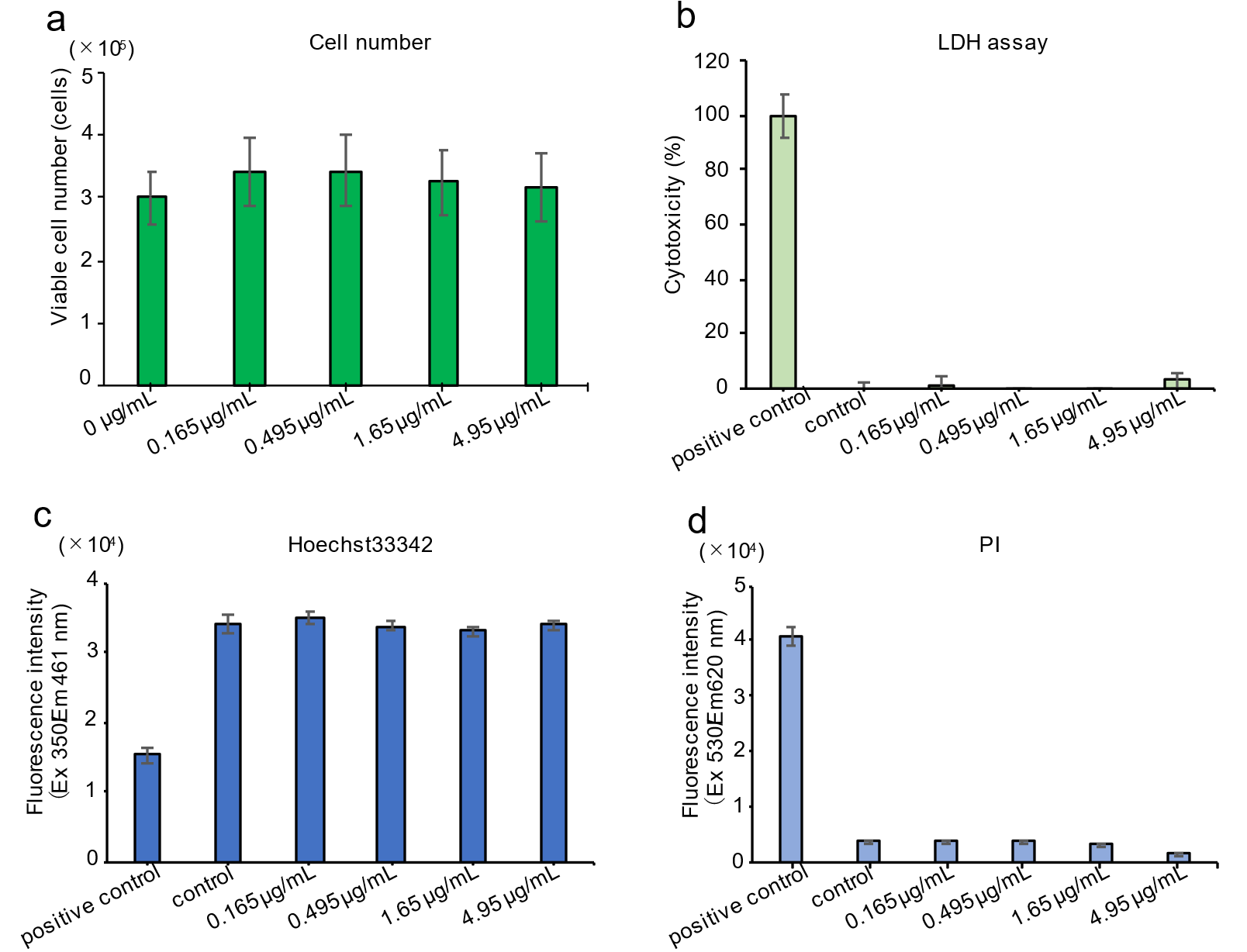


**Supplementary Figure S2.** Effects of *Chlorella* extracts on TIG-118 fibroblast cell viability and death.

For cell viability (a), TIG‐118 cells were seeded in 24 well plates at 2.5×10^5^ cells /well. For LDH assay (b) and hoechst33342/PI (propidium iodide) double staining (c, d), TIG‐118 cells were seeded in 96 well black plates at 5×10^4^ cells/well. Fibroblast cells were treated with CE (0.165 μg/ml; 0.1 μM lutein, 0.495 μg/ml; 0.3 μM lutein, 1.65 μg/ml; 1 μM lutein, and 4.95 μg/ml; 3.0 μM lutein) for three days. For cell number counting (a; n=3), trypan blue (Nacalai tesque Inc., Kyoto, Japan) was used, and only viable cells were counted. For LDH assay (b; n = 3), the cell culture supernatant was collected and measured by Cytotoxicity LDH Assay Kit-WST (DOJINDO LABORATORIES, Kumamoto, Japan) according to the manufacturer’s instructions. For hoechst33342/PI (DOJINDO LABORATORIES, Kumamoto, Japan) double staining, cells were stained with Hoechst and PI for 20 minutes and the fluorescence was measured (d; n = 3). Positive control; cell lysis buffer. Each value represents the mean ± SD.


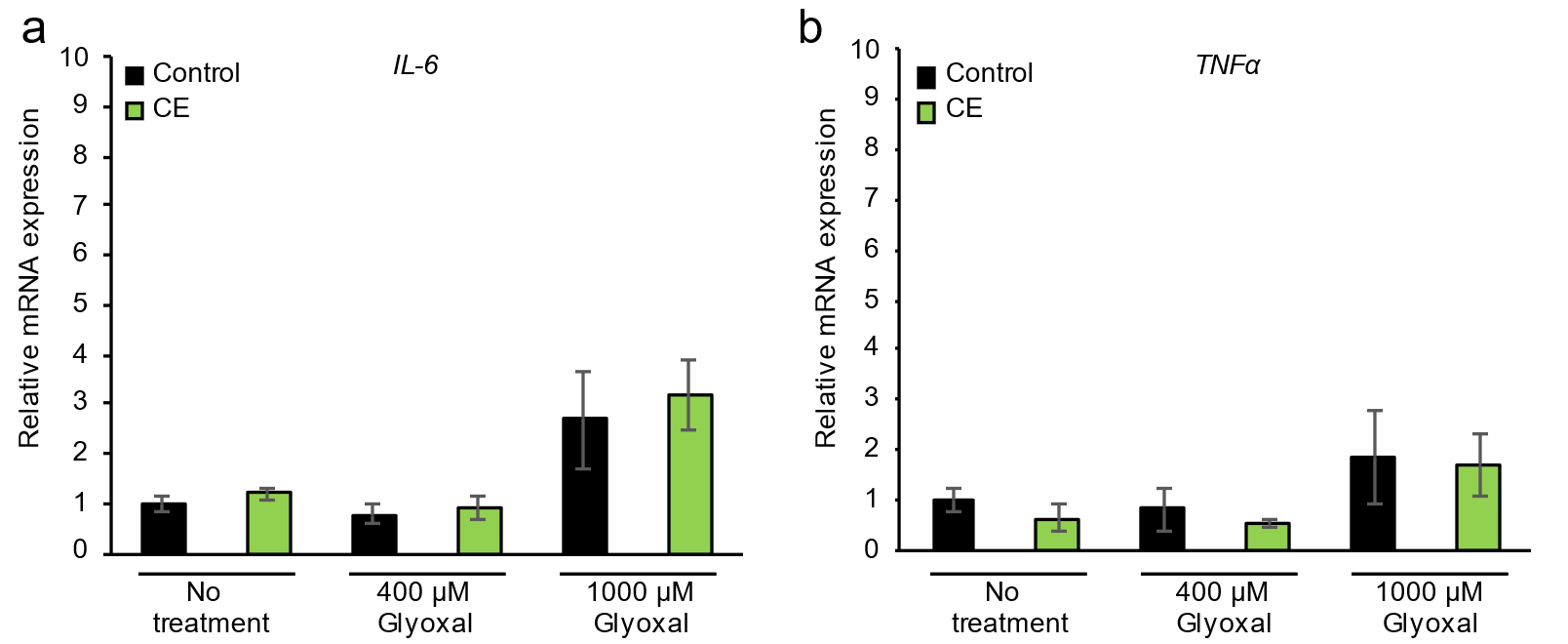


**Supplementary Figure S3.** Effect of *Chlorella* extracts on interleukin 6 (*IL-6*) and tumor necrosis factor alpha (*TNF-α*) expression in the glycated collagen gels. Collagen gels were incubated with 0, 400, or 1000 μM glyoxal for 24 h, and *IL-6* expression (a; n = 3) and *TNF-α* expression levels (b; n = 3) were measured using RT-PCR. CE was administered at 1.65 μg/mL. Each value represents the mean ± SD.
